# Why the processing of repeated targets are better than that of no repetition: evidence from easy-to-difficult and difficult-to-easy switching situations

**DOI:** 10.1186/1744-9081-10-4

**Published:** 2014-02-13

**Authors:** Guangheng Dong, Hongli Zhou, Xiao Lin, Yanbo Hu, Qilin Lu

**Affiliations:** 1 Department of Psychology, Zhejiang Normal University, 688 Yingbin Road, Jinhua, Zhejiang Province, P.R. China; 2 Center for Integrative Neuroscience and Neurodynamics (CINN), School of Psychology and Clinical Language Sciences, University of Reading, Reading, UK; 3 School of Life Science, University of Science and Technology of China, Hefei, Anhui Province, PR China

**Keywords:** Switch cost, Priming effect, Switching situation, Repeating situation

## Abstract

**Background:**

Previous studies have found that the processing of repeated targets are easier than that of non-repetition. Although several theories attempt to explain this issue, the underlying mechanism still remains uncovered. In this study, we tried to address this issue by exploring the underlying brain responses during this process.

**Methods:**

Brain activities were recorded while thirty participants performing a Stroop task (Chinese version) in the MRI scanner. Using pseudo-random strategies, we created two types of switching conditions (*easy-to-difficult*; *difficult-to-easy*) and relevant repeating conditions.

**Results:**

The results show that, in difficult-to-easy switching situation, higher brain activations are found in left precuneus than repeating ones (the precuneus is thought related with attention demands). In easy-to-difficult switching conditions, higher brain activations are found in precuneus, superior temporal gyrus, posterior cingulate cortex, and inferior frontal gyrus than repeating trials (most of these regions are thought related with executive function). No overlapping brain regions are observed in con_CON and incon_INCON conditions. Beta figures of the survived clusters in different conditions, correlations between brain activations and switch cost were calculated.

**Conclusions:**

The present study suggests that the feature that response time in switching trials are longer than that in repeating trials are caused by the extra endeavors engaged in the switching processes.

## Introduction

Plenty of studies have found that the current trial performance is influenced by previous one [1-5], and that the processing of repeated targets are better than that of non-repeated ones [6-8]. For example, individuals tend to show shorter response time and higher response accuracy to incongruent trials following incongruent trials (incon_INCON) compared to incongruent trials following congruent trials (con_INCON) [4,5,9]. In addition, response times for congruent trials following congruent trials (con_CON) tend to be shorter than those for congruent trials followed by incongruent trials (incon_CON) [10,11].

Several theories contributed to understand how previous processed task affect the processing of subsequent stimuli: mental set switching, conflicting adaption, and priming effect. Mental set switching (also termed “mental switch”, “mental shifting”) has been reported to be involved in nearly any type of cognitive switches [12-14]. It is required when the focus of attention must be altered in order to adapt to a frequently changing environment. It is generally assumed that the act of switching is sub-served by a set of executive control parameters necessary to complete the task [15]. When we switch from one type of task to another, extra executive endeavors should be involved to complete this process. The underlying mechanism behind this is the ability to update the executive control parameters representing a given 'task set’ to accommodate a new task set [15,16].

Cognitive adaption refers to the ability to monitor behavior and identify situations that require compensatory adjustments in cognitive resources. Conflicting adaption theory suggests that the conflict triggers the allocation of cognitive resources [5,17]. This theory believe that the phenomenon is due to switching between congruencies and because conflict-driven control reduces the facilitating effect of consecutive repetition of congruent trials [18]. These changes in performance are frequently referred as conflict adaptation or sequential trial effects. According to the conflict monitoring theory, detection of high conflict on incongruent trials should lead to the recruitment of cognitive resources to enhance subsequent performance [19-21]; however, following a congruent trial, less cognitive control is recruited, often resulting in slower RTs and more errors on the subsequent trial.

The priming effect is defined as the influence of an event (prime) on the response of a subsequent event (target), which refers to the exposure to a stimulus influences the response to a later stimulus [6]. The facilitating effect can be observed when there is a link between prime and target. This effect can be independent of the consciousness of priming information, suggesting that the subliminal activation of concepts can effectively influence the judgment and behavior of individuals [22]. The facilitating effect is positive priming, which is thought caused by spreading activation [23-25]. This means that the previous stimulus activates parts of a particular representation or association just before carrying out an action or task. The representation is already partially activated when the second stimulus is encountered, so less additional activation is needed for one to become consciously aware of it.

These three theories focus on different aspects of the cognitive functions during the switching or repeating processes. The set-switching theory pays attention to the extra endeavors in switching trials; The priming emphasizes the facilitation of repeating trials; The conflicting adaption theory believe the conflict control reduces the facilitating effect of consecutive repetition, it focuses on the interaction between facilitating effect and switching cost (extra executive control). Although all of these theories sound reasonable, however, we still don’t know which is right or which is better?

In this study, we tried to address this question by exploring the underlying neural activations during this process. Firstly, different brain networks are involved in different theories. Meta-analyses of relevant functional neuroimaging literatures have confirmed the importance of brain activities within the frontal and the parietal cortex regions when a mental set switch is required [12,26]. These regions include the executive control related brain regions, such as anterior cingulate cortex [27-30], temporal cortex, and the striatum [16,31,32]. However, in priming effect, the middle temporal gyrus and the middle frontal gyrus are typically found engaged in priming processing [33-35]. Thus, we can better understanding the proposed question by comparing activated brain regions during this process.

In addition, task switching theory suggests that people need more endeavors to finish the switching process (switch cost). Thus, the switching trials should engage higher brain activities than repeating trials because they engaged more endeavors in the switching process. However, according to the priming and the facilitation effect, the previous task may triggers network of the following task, which facilitates the processing of current task. Thus, the priming theory and conflict adaption theory predict that a repeated trial would be associated with diminished activations in the same brain regions that were engaged in prior trial. Therefore we can try to distinguish how well different theories explain this issue by comparing the levels of brain activations of different conditions.

## Methods

### Participant selection

All subjects provided written informed consent and thirty subjects (22.4 ± 2.8 years; 5 females) participated. The experiment conforms to The Code of Ethics of the World Medical Association (Declaration of Helsinki). The Human Investigations Committee of Zhejiang Normal University approved this research. All subjects underwent structured psychiatric interviews (M.I.N.I.) [36] performed by an experienced psychiatrist and no active Axis I disorders were present. Depression was further assessed using the Beck Depression Inventory [37] with and exclusionary cut-off of 5. All subjects are right handed and do not suffered head injury with lost consciousness during their lifetime.

### Task and procedure

**Figure 1:**
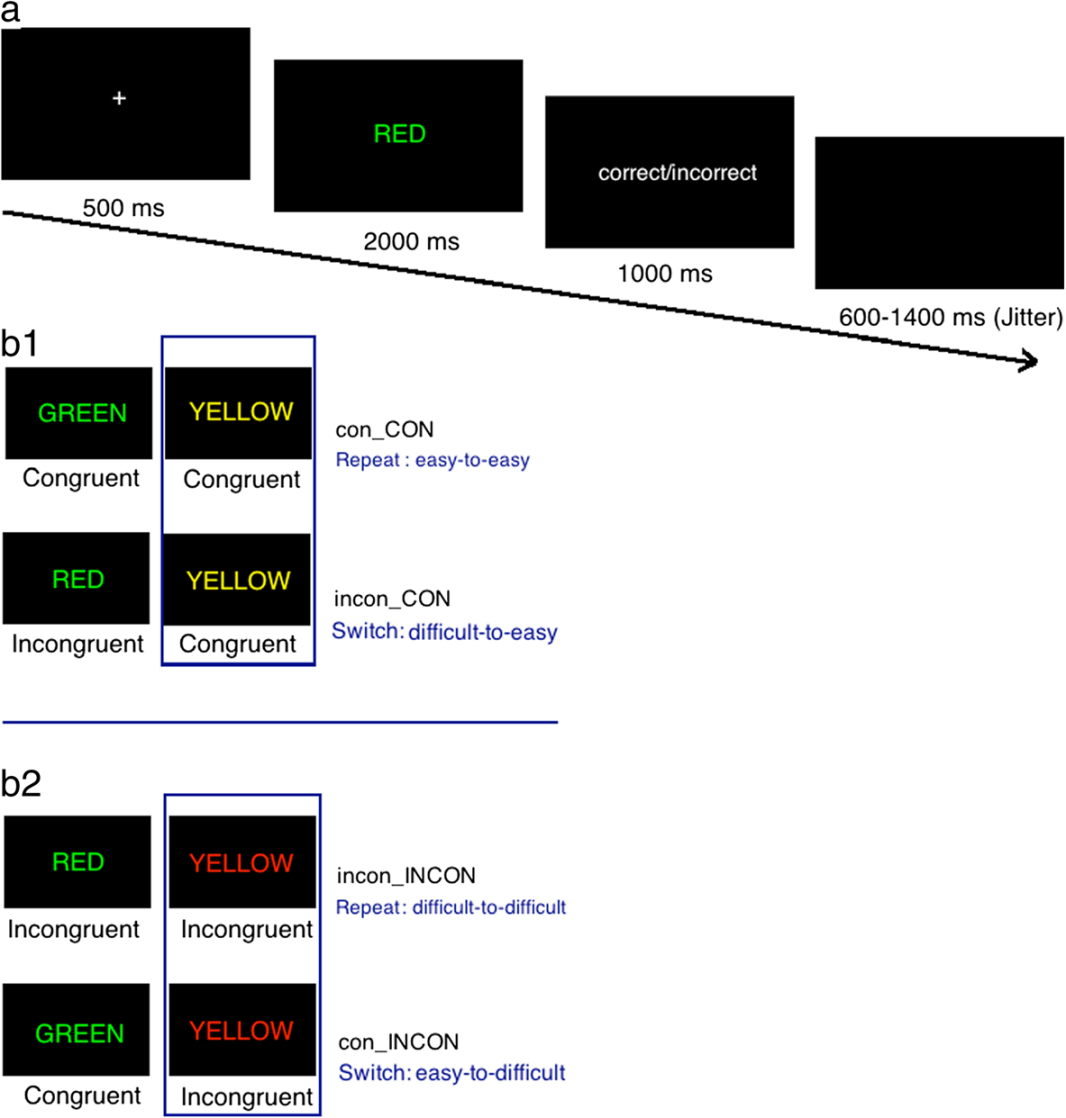
**The timeline of one trial and four conditions we created in present study. a**, The timeline of one trial in the Stroop task in present study. The original version of the Stroop task is in Chinese. The English description is to make readers understand this design easily. **b**, Four conditions we created by manipulating the sequence of congruent and incongruent trials in present study.

An event-related color-word fMRI Stroop task was used in this study. The original version of the Stroop task is in Chinese. Here the English version is to make readers understand this design easily. First, a fixation in the center of the screen (+) lasts for 500 ms. Then, one of the three target color words (e.g. red, green, yellow) was presented in congruent (e.g., the word “RED” in red ink) or incongruent (e.g., the word “RED” in green ink) trials. After 2000 ms, the feedback screen will last for 1000 ms. The task was comprised of 2 sessions of 120 trials each. Each trial was presented for 2000 ms. Participants were asked to press a button to indicate to the ink color of the word as soon as possible using three buttons (i.e., green = thumb, red = index finger, yellow = middle finger; counter-balanced between subjects) of a five-button response box (Invivo Corp.; http://www.invivocorp.com/). A black screen was presented for a random interval of 600–1400 ms (average 1000 ms) between trials [38] (Figure 1a). The probability of congruent and incongruent trials is 50%: 50%. Stimuli were presented and behavioral data were collected using E-prime software (Psychology Software Tools, Inc.). Participants were told that they would be paid a guaranteed 50 Yuan (≈8 US$) for participation and, to encourage quick and accurate task performance, were told they would be rewarded with an additional 0–50 Yuan based on their task performance [1/(reaction time * error rate)]. Participants completed an out-of-scanner practice session which continued until they reached an accuracy rate of 90% or higher.

Although participants were told to perform a totally randomized task, in fact, all trials were predetermined (pseudo-random design). We created four types of conditions with this method: (1) Congruent trial following congruent trial (con_CON); (2) Congruent trial following incongruent trial (incon_CON); (3) Incongruent trial following incongruent trial (incon_INCON); (4) Incongruent trial following congruent trial (con_INCON) (Figure 1b).

There are totally 240 trials in the whole task. Each trial, except the last one, was followed by another trial. In order to create more switching/repeating trials, the number of trials of congruent/incongruent was 120:120, which is a bit different from classical Stroop tasks. Using this method, we created 239 switching trials in four conditions. Thus, each condition consists about 60 trials. In fact, a pseudo-random is not a must because the totally randomized task can also create the same number of switching/repeating trials. However, in order to make the data analysis easier, we used pseudo-random design.

### Image acquisition and pre-processing

Structural images covering the whole brain were collected using a T1-weighted three-dimensional spoiled gradient-recalled sequence (176 slices, TR = 1700 ms, TE = 3.93 ms, slice thickness = 1.0 mm, skip = 0 mm, flip angle = 15°, inversion time 1100 ms, field of view = 240*240 mm, in-plane resolution = 256*256). Functional MRI was performed on a 3 T scanner (Siemens Trio) with a gradient-echo EPI T2 sensitive pulse sequence in 33 slices (interleaved sequence, 3 mm thickness, TR = 2000 ms, flip angle 90°, field of view 220 × 220 mm^2^, matrix 64 × 64). Stimuli were presented using Invivo synchronous system (Invivo Company, http://www.invivocorp.com) through a screen in the head coil, enabling participants to view the stimuli.

### First-level regression analysis

Imaging analysis was conducted using SPM5 (http://www.fil.ion.ucl.ac.uk/spm). Images were slice-timed, reoriented, and realigned to the first volume. T1-co-registered volumes to correct for head movements. Images were then normalized to an MNI space (defined by Montreal Neurological Institute) and spatially smoothed using a 6 mm FWHM Gaussian kernel. A general linear model (GLM) was applied to identify blood oxygen level dependence (BOLD) activation in relation to separate event types. There were four types of trials: incon_CON, incon_INCON, con_INCON, and con_CON. Six head-movement parameters derived from the realignment stage were included to exclude motion related variances. GLM was independently applied to each voxel to identify voxels that were significantly activated for the each event that was modeled. All incorrect answers will be excluded from further analysis.

### Second-level analysis

Second level analysis treated inter-subject variability as a random effect. First, we determined voxels showing a main effect in different conditions relative to implicit baseline. Second, we tested for voxels that showed higher or lower activity in two contrasts of interest (difficult-to-easy: incon_CON > con_CON; easy-to-difficult: con_INCON > incon_INCON). We first identified clusters of contiguously significant voxels at an uncorrected threshold *p* < 0.01, as also used for display purposes in the figures. We then tested these clusters for cluster-level FWE correction *p* < 0.01 and the AlphaSim estimation indicated that clusters with 30 contiguous voxels would achieve an effective FWE threshold *p* < 0.01. The smoothing kernel used during simulating false-positive (noise) maps using AlphaSim was 6.0 mm, and was estimated from the residual fields of the contrast maps being entered into the one-sample *t*-test. The formula used to compute the smoothness is that used in FSL (see http://www.fmrib.ox.ac.uk/analysis/techrep/tr00df1/tr00df1/node6.html for more information).

### Correlation analysis

Correlation analysis was calculated between brain activities and the behavioral performances to support our hypothesis. We used peak beta values of the survived clusters as the index of brain activities in related brain regions. First, the correlation between precuneus activation and the switch cost (RT in con_INCON > incon_INCON, incon_CON > con_CON). Second, the correlations between brain activations in superior temporal gyrus and posterior cingulate cortex and the switch cost (RT in con_INCON > incon_INCON) in easy-to-difficult switching trials.

## Results

### Behavioral performance

First, significant Stroop effect was observed when comparing incongruent trials to congruent tirals [*t* = 7.621, *p* < 0.001], which proved that the Stroop task is valid in current study. A repeated-measures ANOVA (current trial type (CON, INCON) * previous trial type (con, incon)) indicated a significant effect of condition on response time (RT) [*F *(3,29) = 34.879, *p* = 0.00]. Post-Hoc analysis (LSD) revealed that both switching situations show longer RT than repeating situations: incon_CON > con_CON [*F *(1,29) = 21.318, *p* = 0.00], and con_INCON > incon_INCON [*F* (1,29) = 56.109, *p* = 0.00] (Table 1). Significant difference was also found when comparing incon_CON to con_INCON [*F* = 4.312, *p* < 0.05], but this was out of our research interest. In error rates, most participants reached a very high accuracy (Mean = 98.3%), thus, we did not compare these results in present study.

**Table 1:** Behavioral performance among different comparisons

	***RT (Mean)***	***SD***	***F***	***p***
con-CON	613.61	130.556	21.3	.000
incon-CON	715.14	158.333		
incon-INCON	649.16	140.952	56.109	.000
con-INCON	747.14	172.656		

The RT in different conditions is the second trial (the capital one) during the switching/repeating situation.

### Imaging results

#### Difficult-to-easy (incon_CON > con_CON)

**Figure 2:**
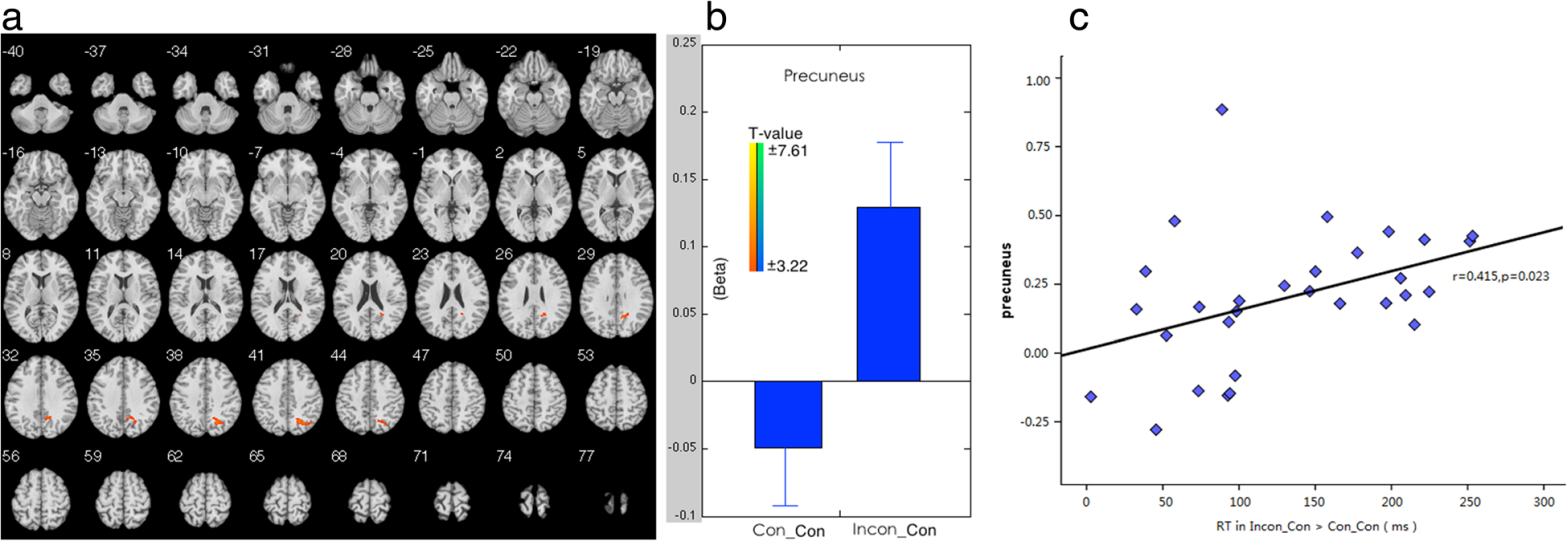
**Imaging results in difficult-to-easy switching situation. a**, The imaging results showed that the switching condition elicited higher brain activation in right precuneus. **b**, Beta figures of the survived cluster in different conditions. **c**, Correlation between brain activations in precuneus and switching cost (RT in Incon_Con > Con_Con).

In congruent trials, the switching situation (incon_CON) elicited higher brain activation in left precuneus than repeating trials (con_CON)(Table 2, Figure 2a). Beta figure showed that the difference was caused by the enhanced brain activations in switching situations (incon-CON) (Figure 2b).

**Table 2:** Regional brain activity changes in different comparisons

**x,y,z**^**a**^	**Hemisphere**	**Peak intensity**	**Number of voxels**^**b**^	**Region**^**c**^	**Brodmann’s area**
Incon_Con > Con_Con (Lower activated)					
24 -63 39	R	3.817	34	Precuneus	7, 31
Con_Incon > Incon-Incon (Higher activated)					
-63 -18 -6	L	7.604	47	Superior_Temporal_Gyrus	39
-3 -30 36	L	4.112	39	Precuneus, Angular gyrus	7,31
-39 51 6	L	5.367	170	Middle_Frontal_Gyrus	46
-33 18 30	L	4.992	89	Middle_Frontal_Gyrus	9
33 39 39	R	4.241	53	Middle_Frontal_Gyrus	9
33 -9 39	R	3.992	35	Posterior_Cingulate_Cortex	31
15 48 -15	R	3.936	48	Superior_Frontal_Gyrus	11
48 33 18	R	5.270	212	Inferior_Frontal_Gyrus	8,9,46

^a^Peak MNI Coordinates.

^b^We first identified clusters of contiguously significant voxels at an uncorrected threshold *p* < 0.01, as also used for display purposes in the figures. We then tested these clusters for cluster-level FWE correction *p* < 0.01 and the AlphaSim estimation indicated that clusters with 30 contiguous voxels would achieve an effective FWE threshold *p* < 0.01. Voxel size = 3*3*3.

^c^The brain regions were referenced to the software Xjview (http://www.alivelearn.net/xjview8) and double checked with atlas.

#### Easy-to-difficult (con-INCON > incon-INCON)

**Figure 3:**
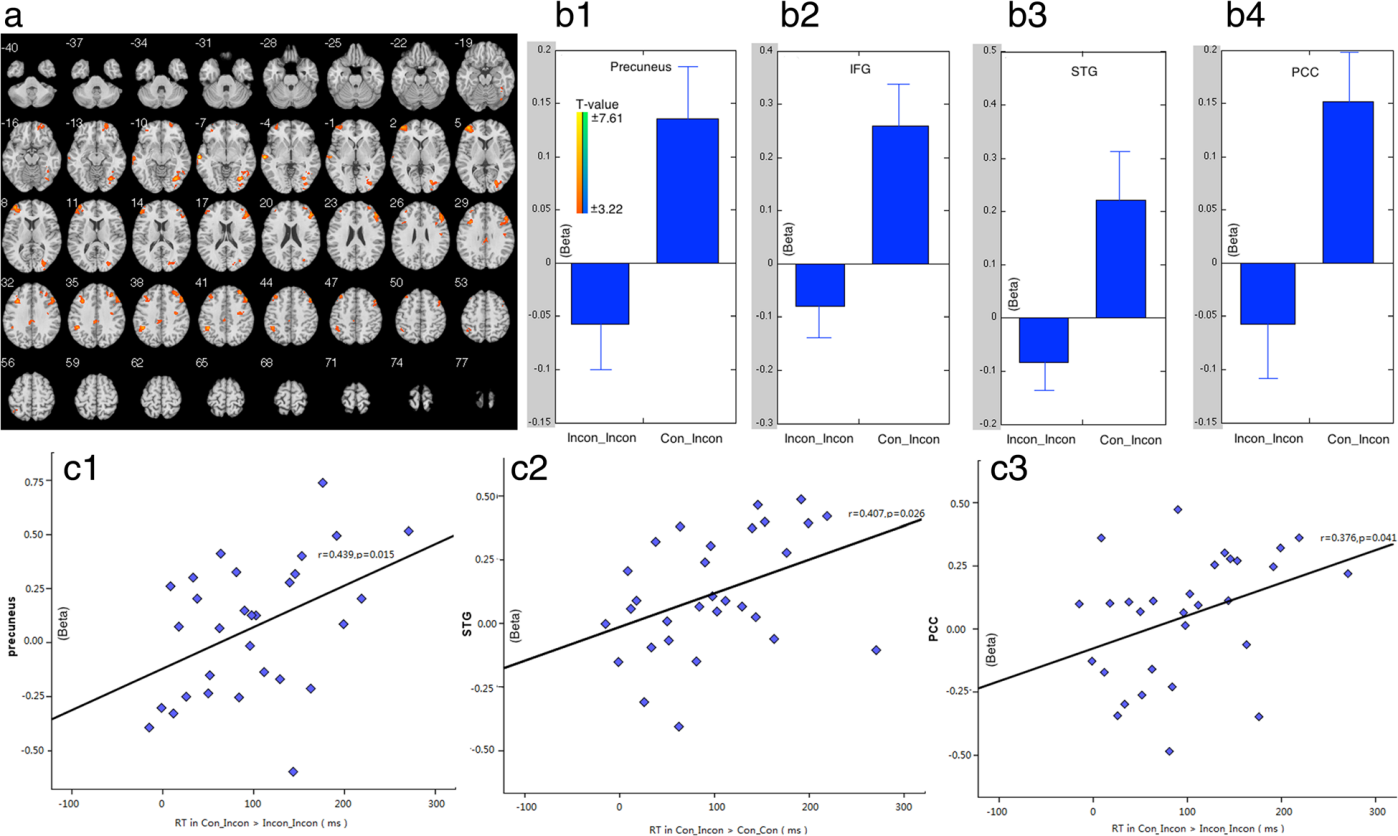
**Imaging results in easy-to-difficult switching situation. a**, Survived clusters when comparing Con_Incon to Incon_Incon conditions. **b**, Beta figures of the survived clusters in different conditions. **c**, Correlation between survived clusters in the comparision and switching cost (RT in Con_Incon > Incon_Incon).

In incongruent trials, the switching situation (con-INCON) brought high brain activations in precuneus and superior temporal gyrus (STG), bilateral middle frontal gyrus, bilateral posterior cingulate cortex (PCC), right superior frontal gyrus, and right inferior frontal gyrus (IFG) than repeating trials (Table 2; Figure 3a). Beta figures showed that all of these differences were caused by the enhanced brain activations in switching siutations (con_INCON) (Figure 3:b1, b2, b3, b4).

### Correlation results

First, significant correlation was found between the brain activity in precuneus (peak Beta value) and the switch cost in con_INCON > incon_INCON (*r* = 0.439, *p* = 0.015), and incon_CON > con_CON (*r* = 0.415, *p* = 0.023) (Figure 2c, 3c1). Significant correlations were also found between brain activations in STG (*r* = 0.407, *p* = 0.026) and PCC (*r* = 0.376, *p* = 0.041) and the switch cost (RT in con_INCON > incon_INCON) in easy-to-difficult switching trials (Figure 3:c2, c3).

#### Brain activations in con_CON and incon_INCON

**Figure 4:**
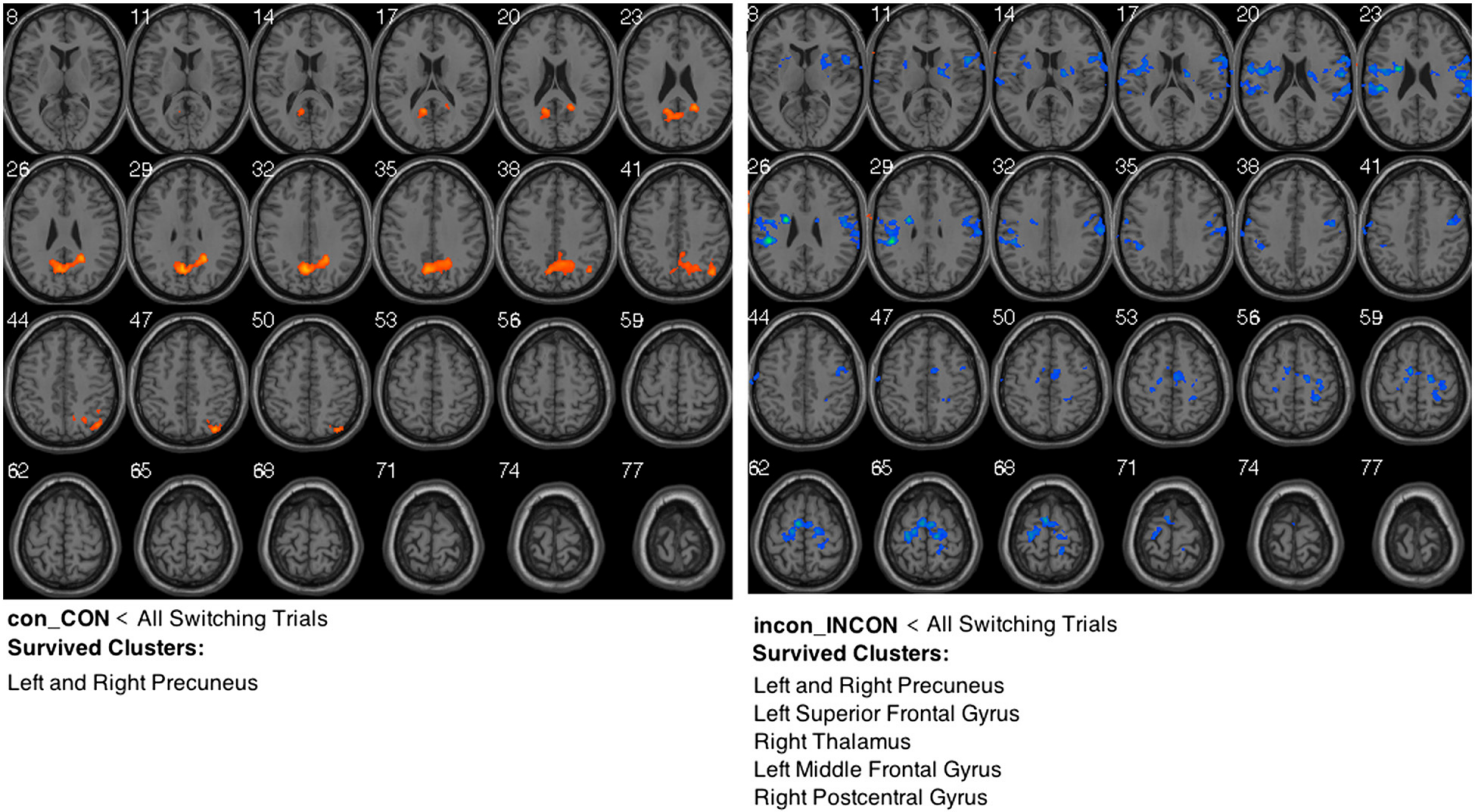
Brain activations in con_CON and incon_INCON conditions in this study.

To find the potential priming or facilitating effect that might hide behind the executive function, we observed the brain activations in con_CON < all switching trials and incon_INCON < all switching trials, separately. The logic is that if there are overlapping brain regions that activated in both conditions, and the brain regions show increase/decrease activation within repeat/switching condition. That can prove that priming effect involved/excluded in this task. Figure 4 shows the brain regions that survived in different comparisons: There is no overlapping brain regions survived in these two conditions. In addition, previous priming studies showed that the middle temporal gyrus and the middle frontal gyrus are typically engaged in this process [33-35]. However, from the survived clusters in different comparisons, we cannot find relevant brain regions activated in this study. All these results might suggest that no priming effect exist during this process.

## Discussion

### More attention engaged in switching trials

Higher precuneus activations were found in both switching situations (incon_CON; con_INCON) when comparing with relevant repeating ones. The precuneus is near the superior parietal area [39] implicated in the control of attentional switching, and switching attention between visual attributes [40]. Previous studies have found that the precuneus activities reflect increased visual attention due to more difficult task demands [41,42]. Studies on cognitive inflexibility found lower precuneus activities in major depression subjects which reflected deficits in attention control [41]. Astafiev found that the precuneus was more active in challenging tasks than in simple tasks [43]. All these results suggest that precuneus activities increase with the increase of attentional demands. In this study, the correlations between precuneus activations and the switch cost in different switching situations also support this conclusion. Take the behavioral and imaging results into consideration, we conclude that the switching conditions recruited more attention than repeating ones in this study.

### More executive control during switching trials

In incongruent trials, the switching situation elicited higher brain activations in left STG, bilateral PCC, and right IFG. All of these brain regions are thought related with executive control.

The STG was found activated during task shifting [26,44], which is supposed to be responsible for inhibitory control in Stroop task. The STG has been found activated when inhibitory control was applied [27,45-47]. Furthermore, studies found that cocaine-dependent patients show lower gray matter density in STG than healthy controls [48,49]. To make sure if the STG activations were caused by inhibitory process in this study, we performed correlation analyses between STG activations and Stroop effects. The correlations in different switching situations support the conclusion that the STG activations are related with executive function in this study.

PCC is a central node of the default mode network - a set of brain regions that show strongly correlated neural activity and reliable deactivation in activity during many cognitive tasks [50,51]. PCC serves multiple roles, including an active role in the regulation of cognition and in cognitive control [52-54]. To determine whether PCC activation is related to executive control in this study, we performed the correlation analysis between the Stroop effect and activation in PCC. The significant correlation between these two measurements also supports the hypothesis.

The right IFG has been typically implicated in inhibitive tasks [55]. The right-IFG (but not left-IFG) damage in humans crucially affects inhibition and task switching [56,57]. Neuroimaging studies of inhibition found bilateral IFG activation [58,59], as do studies of switching or shifting [60,61]. The damage of the right IFC impairs independent measures of executive control by disrupting inhibition (specifically of responses and task-sets) [55].

Take all these results into consideration, we conclude that participants engaged more executive control in the switching situation than in repeating situation.

### No priming or facilitating effect was found in repeating trials

When we observing the brain activations in con_CON and incon_INCON conditions, no common brain regions were found in these two conditions, even the features of activation were totally different in different conditions (in con_CON, higher activation were found in precuneus; in incon_INCON, lower brain activation were found in some brain regions). In addition, previous priming studies showed that the middle temporal gyrus and the middle frontal gyrus are typically engaged in this process [33-35]. However, from the survived clusters in different comparisons, we did not find relevant brain regions activated in this study. Thus, from what we got, there is no priming effect observed during this process.

### The possible underlying mechanism

First, priming effect was not found when observing the brain activations in con_CON and incon_INCON conditions. Which might suggest that priming effect is short of solid support in this study. Second, the switching conditions (difficult-to-easy and easy-to-difficult) recruited more attention and engaged more executive function than repeating ones, which can be indexed by the heightened brain activations in related brain regions. Third, the conflicting adaption theory believes that the switching between congruent and incongruent trials reduces the facilitating effect of consecutive repetition of congruent trials [2,18]. This theory paid attention to both of the facilitating effect and the endeavors engaged in executive control. Because there is no priming of facilitating effect found in this study, the current results only support part of the conflicting adaption theory.

From what we discussed above, the present study suggest that the feature that response time in switching trials are longer than in repeating trials are caused by the extra endeavors engaged in the switching processes.

### Limitations

There are several limitations should be regarded here. First, since there are only 5 females and 25 males participated this study. The imbalance in gender might limit the value of the final conclusion. Second, The brain regions that activated by different switching tasks vary a lot. It is really hard to find overlapped brain regions that activated by each of these tasks and been replicated repeatedly. This study relies on inverse inference in some degrees in interpreting the brain activations during this process. Third, although there is no priming effect detected in this study, however, an alternative explanation is that the priming effect is fast and implicit, and it is hard to be detected by fMRI techniques. Fourth, the fast changing Stroop task might has limitation in detecting priming effect; future studies should try to explore this issue with other paradigms.

## Competing interests

The authors have declared that no competing interests exist.

## Authors’ contributions

Conceived and designed the experiments: GD, HZ. Performed the experiments: GD, XL. Analyzed the data: GD, XL, QL. Contributed reagents/materials/analysis tools: GD XL. Wrote the paper: GD, YH, HZ. All authors read approved the final manuscript.
